# A Guide to Surgical Diagnosis

**Published:** 1879-10

**Authors:** 


*A Guide to Surgical Diagnosis.* By Christopher Heath, F. R. C. S., Prof. of Clinical Surgery in University College, London, etc. Publishers: Lindsay & Blakiston, Philadelphia, 1879.

The mere announcement of a work by this celebrated Surgeon is a sufficient recommendation of its value, and the present one is no exception to the rule, for his many years' experience as a clinical teacher, renders him authority on all such subjects. In the present work, which is a most useful one to the practitioner as well as to the student, the surgical affections are arranged anatomically, in order to assist the reader in obtaining a clue to the nature of such cases where the advantages of the presence of the patient may be absent. Without any attempt being made to discuss the pathology and treatment of any of the diseases described, yet the salient points receive due notice, and the differential diagnosis of affections likely to be confounded, is plainly pointed out. The work commences with "Heads for Report on Surgical Cases used in the University College Hospital, London, compiled by Mr. Godlee; and the text proper, with Affections of the Head." The Affections of the Mouth follow, including those of the lips, tongue, jaws, and gums. Affections of all the other parts of the body receive due notice in regular anatomical order, rendering this work an exceedingly useful one, in which a great deal of information is condensed into a small volume.

